# Supplementary material for: Derivation of human primordial germ cell-like cells in an embryonic-like culture
Source: Nat Commun. 2024 Jan 2;15:167. doi: 10.1038/s41467-023-43871-2 (PMC10762101; doi:10.1038/s41467-023-43871-2)
Supplement: Supplementary file 1 — Supplementary Information [file 41467_2023_43871_MOESM1_ESM.pdf]

Derivation of Human Primordial Germ Cell-Like Cells in an Embryonic-Like Culture

Sajedeh Nasr Esfahani[1 †], Yi Zheng[1 † #], Auriana Arabpour[2, 3, 4 †], Agnes M. Resto Irizarry[1], Norio Kobayashi[1], Xufeng Xue[1], Yue Shao[5], Cheng Zhao[6], Nicole L. Agranonik[4], Megan Sparrow[4], Timothy J. Hunt[4], Jared Faith[4], Mary Jasmine Lara[4], Qiu Ya Wu[4], Sherman Silber[7], Sophie Petropoulos[6, 8, 9, 10], Ran Yang[11], Kenneth R. Chien[11], Amander T. Clark[2, 3, 4 *], and Jianping Fu[1, 12, 13 *]

[1]Department of Mechanical Engineering, University of Michigan, Ann Arbor, Michigan 48109, USA;

[2]Molecular Biology Institute, University of California, Los Angeles, Los Angeles, California 90095, USA;

[3]Eli and Edythe Broad Center of Regenerative Medicine and Stem Cell Research, University of California, Los Angeles, Los Angeles, California 90095, USA;

[4]Department of Molecular, Cell and Developmental Biology, University of California, Los Angeles, Los Angeles, California 90095, USA;

[5]Institute of Biomechanics and Medical Engineering, Department of Engineering Mechanics, School of Aerospace Engineering, Tsinghua University, Beijing 100084, China;

[6]Department of Clinical Science, Intervention and Technology, Division of Obstetrics and Gynecology, Karolinska Instituet, 14186 Stockholm, Sweden;

[7]Infertility Center of St. Louis, St. Lukes Hospital, St. Louis, Missouri 63017, USA;

[8]Département de Médecine, Université de Montréal, Montréal, Canada;

[9]Centre de Recherche du Centre Hospitalier de l'Université de Montréal, Axe Immunopathologie, H2X 19A Montreal, Canada;

[10]Département de Médecine, Molecular Biology Programme, Université de Montréal, Montréal, Canada

[11]Department of Cell and Molecular Biology, Karolinska Institutet, Stockholm 171 77, Sweden;

[12]Department of Cell & Developmental Biology, University of Michigan Medical School, Ann Arbor, Michigan 48109, USA;

[13]Department of Biomedical Engineering, University of Michigan, Ann Arbor, Michigan 48109, USA.

[†]These authors contributed equally to this work. *Correspondence and requests for materials should be addressed to J.F. (email: jpfu@umich.edu) and A.T.C. (email: clarka@ucla.edu).

[#]Present address: Department of Biomedical and Chemical Engineering, Syracuse University, Syracuse, NY 13244, USA.

Supplementary Figures

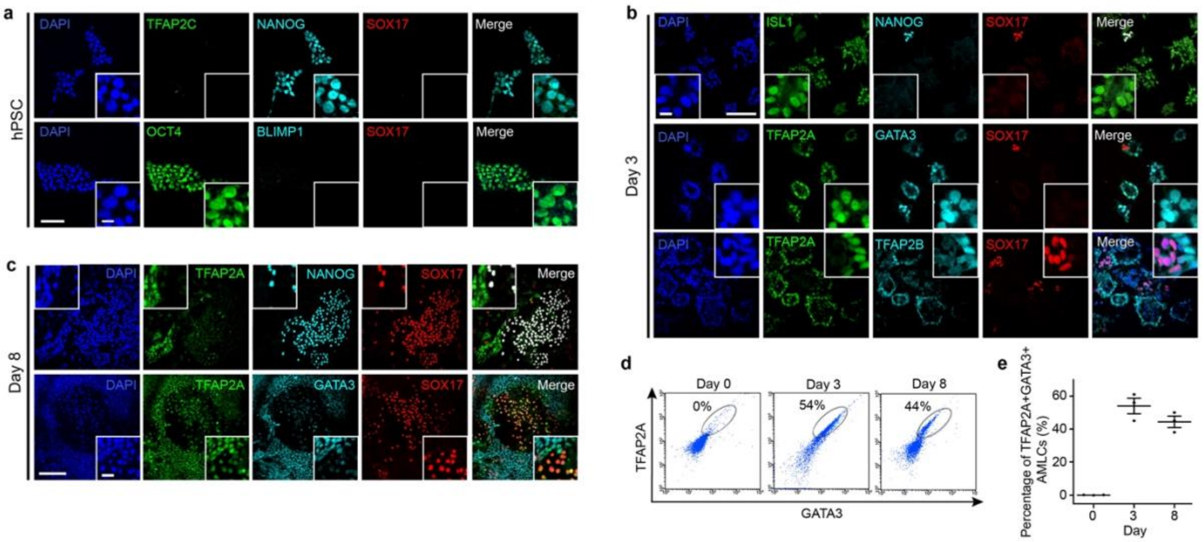

Supplementary Figure 1: Characterization of the Gel-3D culture for hPGCLC induction.

- **a.** Micrographs showing immunostaining for TFAP2C, NANOG and SOX17 (top); OCT4, BLIMP1, and SOX17 (bottom) in hPSCs.
- **b.** Micrographs showing immunostaining for ISL1, NANOG, and SOX17 (top); TFAP2A, GATA3, and SOX17 (middle); and TFAP2A, TFAP2B, and SOX17 (bottom) in the Gel-3D culture on Day 3.
- **c.** Micrographs showing immunostaining for TFAP2A, NANOG, and SOX17 (top); TFAP2A, GATA3, and SOX17 (bottom) in the Gel-3D culture on Day 8.
- **d.** Enumeration of TFAP2A+GATA3+ AMLCs in the Gel-3D culture using flow cytometry at indicated time points.
- **e.** Plot showing percentages of TFAP2A+GATA3+ AMLCs at indicated time points.

In **a-c**, experiments were repeated three times with similar results. Nuclei were counterstained with DAPI. Boxed images show magnified views of selected areas. Scale bars, 200 µm (main

panels) and 30 µm (insets). In **d** & **e**, experiments were repeated three times with similar results.

In **e**, data represent the mean ± s.e.m. Source data are provided as a Source Data file.

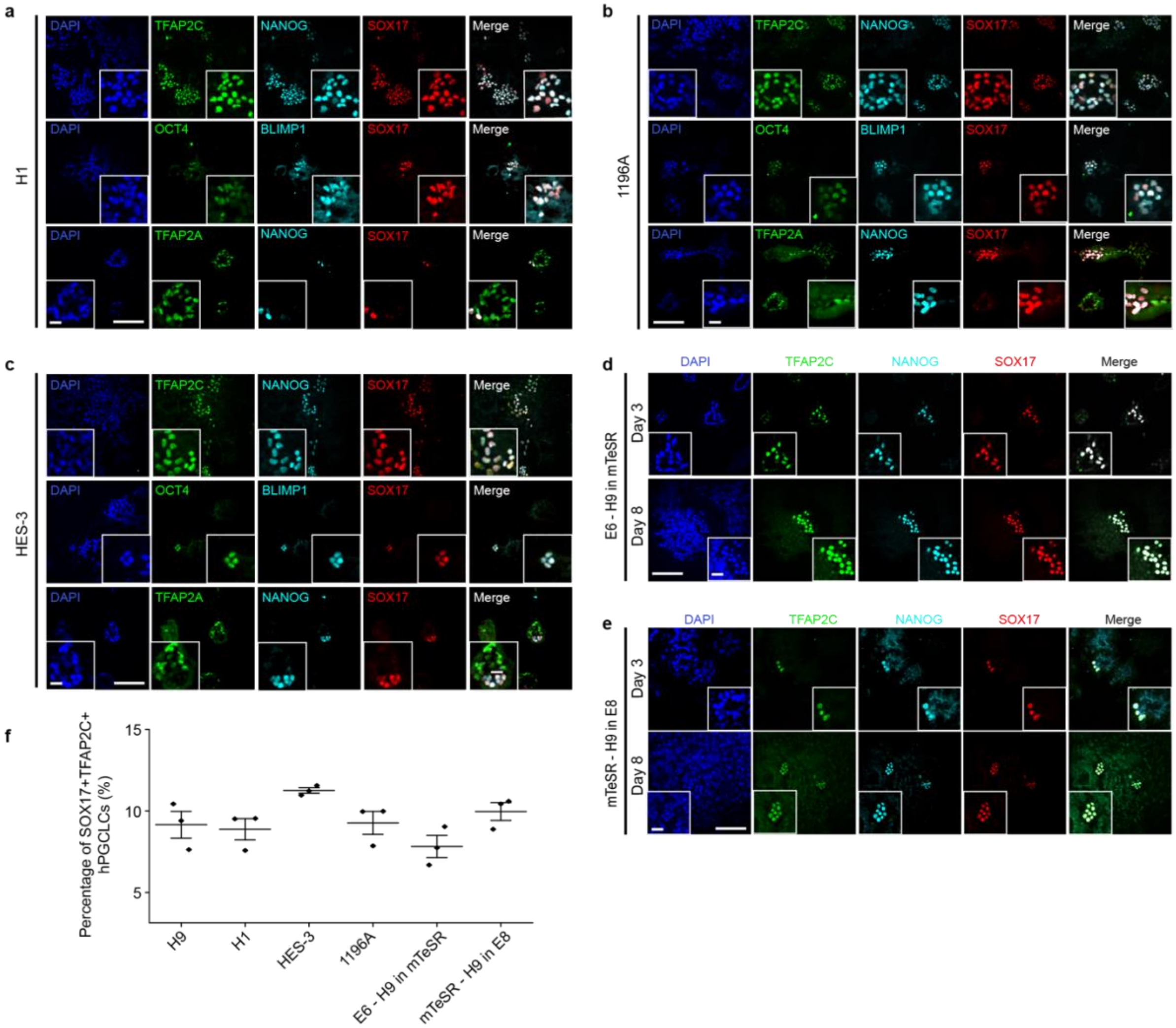

Supplementary Figure 2: hPGCLC induction in the Gel-3D culture generated from different hPSC lines and culture medium conditions.

a-c. Micrographs showing immunostaining for TFAP2C, NANOG, and SOX17 (top); OCT4, BLIMP1, and SOX17 (middle); and TFAP2A, NANOG, and SOX17 (bottom) in the Gel-3D culture on Day 3, using H1 human embryonic stem cells (**a**), 1196A human induced pluripotent stem cells (**b**), and HES-3 human embryonic stem cells (**c**).

d. Micrographs showing immunostaining on Day 3 and on Day 8 for TFAP2C, NANOG, and SOX17 in the Gel-3D culture with Essential 6 as culture medium. H9 hPSCs were maintained in mTeSR.

e. Micrographs showing immunostaining on Day 3 and on Day 8 for TFAP2C, NANOG, and SOX17 in the Gel-3D culture with mTeSR as culture medium. H9 hPSCs were maintained in E8.

f. Plot showing percentages of TFAP2C+SOX17+ hPGCLCs on Day 3 as a function of hPSC lines and culture medium conditions.

In **a-e**, experiments were repeated three times with similar results. Nuclei were counterstained with DAPI. Boxed images show magnified views of selected areas. Scale bars, 100 µm (main panels) and 30 µm (insets). In **f**, n = 3 independent experiments, and data represent the mean ± s.e.m. Source data are provided as a Source Data file.

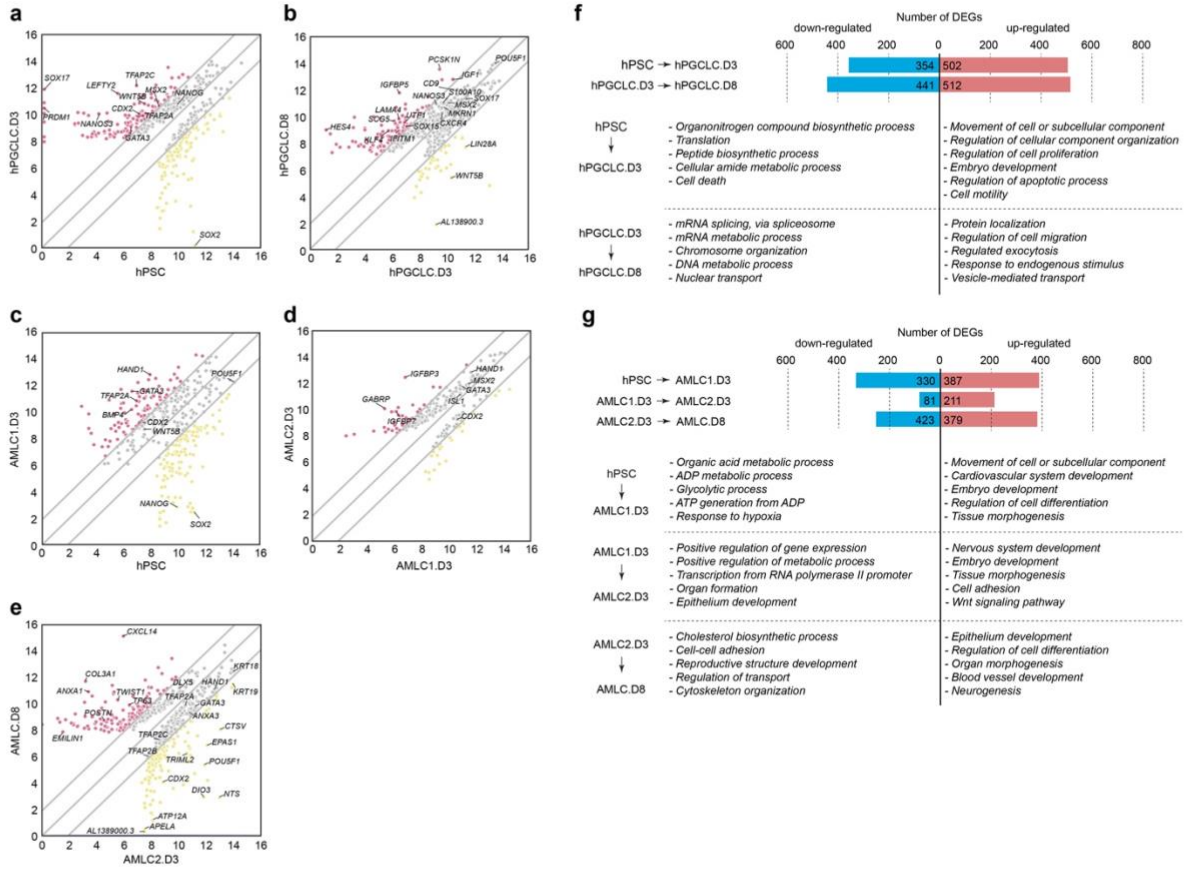

Supplementary Figure 3: Differentially expressed genes (DEGs) during hPGCLC and AMLC lineage development.

a-e. Scatter plots showing expression of DEGs between indicated pairs of cell clusters. Some genes are highlighted as indicated.

f&g. DEGs between different cell clusters as indicated. Top, red and blue bars indicate the numbers of up- and downregulated DEGs, respectively, in indicated pairwise comparisons. Bottom, enrichment of GO terms in DEGs from indicated pairwise comparisons.

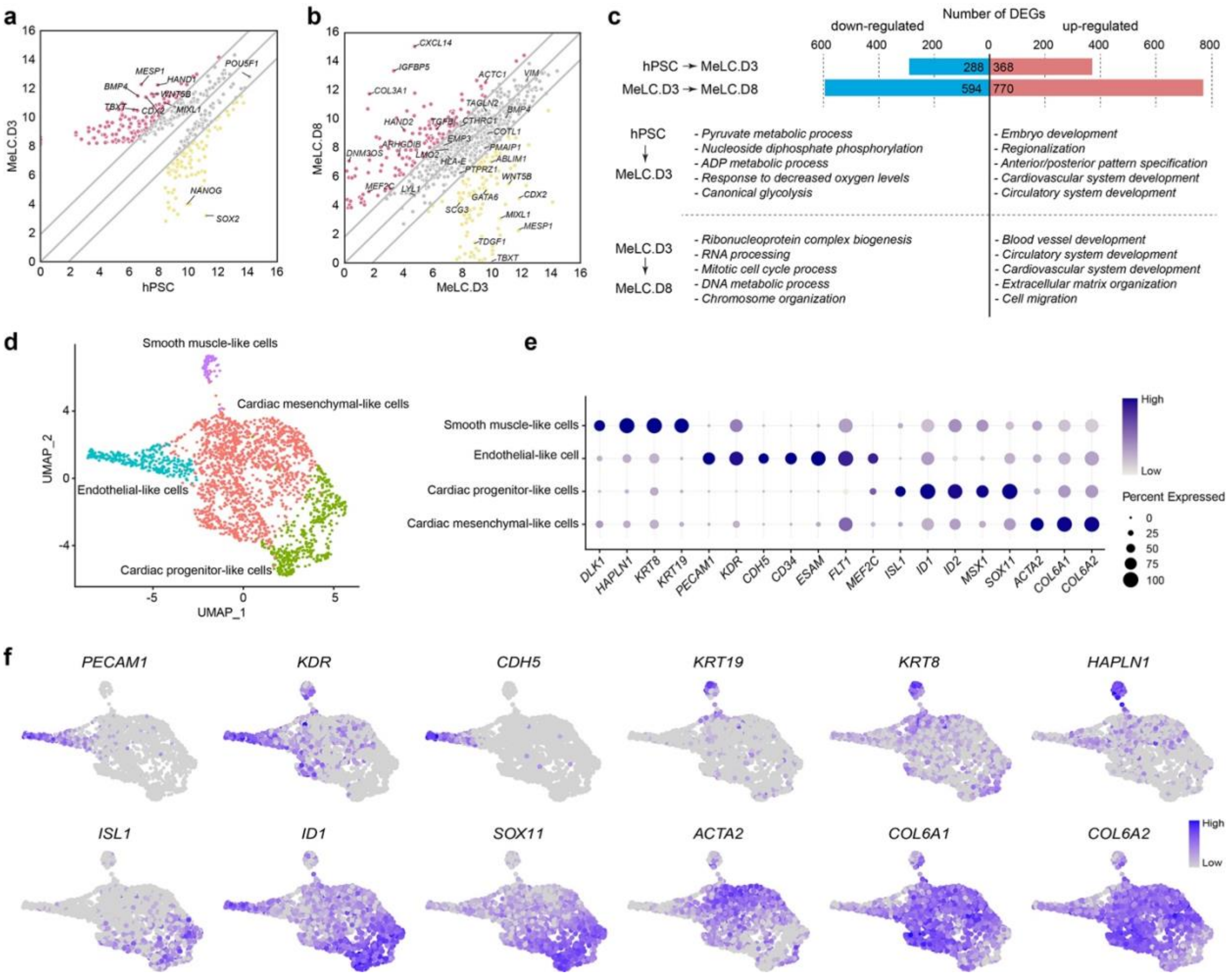

Supplementary Figure 4: Single-cell transcriptomic analysis of MeLC lineage in the Gel-3D culture.

- **a&b.** Scatter plots of expression of DEGs between indicated pairs of cell clusters. Some genes are highlighted as indicated.
- **c.** DEGs between different cell clusters as indicated. Top, red and blue bars indicate the numbers of up- and downregulated DEGs, respectively, in indicated pairwise comparisons. Bottom, enrichment of GO terms in DEGs from indicated pairwise comparisons.

d. UMAP plot generated from scRNA-seq data of Day 8 MeLC cluster, revealing four distinct, color-coded cell populations annotated as cardiac mesenchymal-like cells, cardiac progenitor-like cells, endothelial-like cells, and smooth muscle-like cells.

e. Dot plot showing expression of key marker genes across the cell clusters in the UMAP in **d** as indicated. The sizes and colors of dots indicate the proportion of cells expressing the corresponding genes and their averaged scaled values of log-transformed expression, respectively.

f. Feature plots showing expression of selected cell lineage markers used for cell identity annotations in **d**.

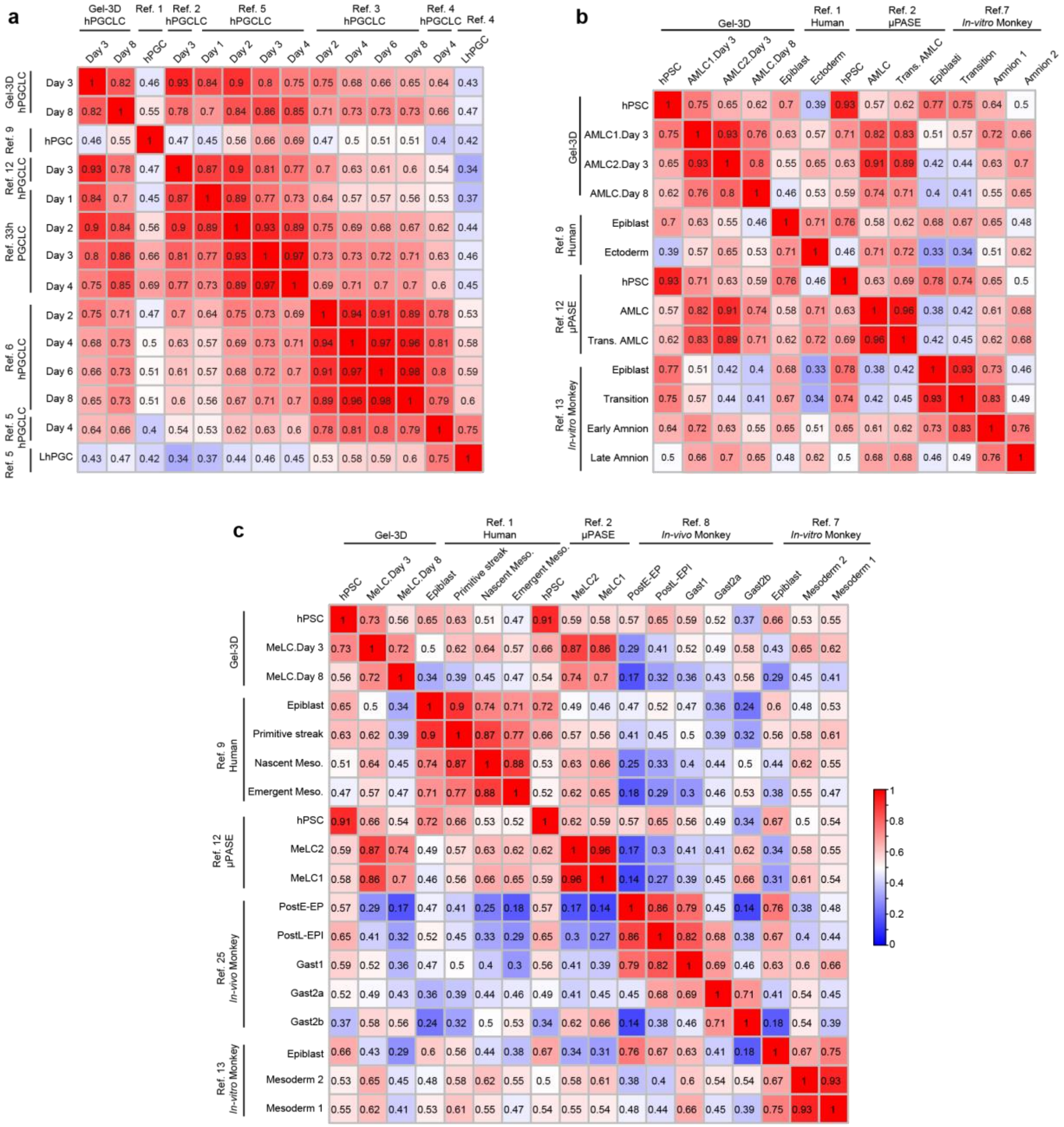
a
Gel-3D hPGCLC
Ref. 1
Ref. 2 hPGCLC
Ref. 5 hPGCLC
Ref. 3 hPGCLC
Ref. 4 hPGCLC
Ref. 4
Day 3
Day 8
hPGC
Day 1
Day 2
Day 4
Day 6
LhPGC
Ref. 9
Ref. 12 hPGCLC
Ref. 33h PGCLC
Ref. 6 hPGCLC
Ref. 5 hPGCLC
Ref. 5
b
Gel-3D
Ref. 1 Human
Ref. 2 µPASE
Ref.7 In-vitro Monkey
hPSC
AMLC1.Day 3
AMLC2.Day 3
AMLC.Day 8
Epiblast
Ectoderm
AMLC
Trans. AMLC
Epiblasti
Transition
Amnion 1
Amnion 2
Ref. 9 Human
Ref. 12 µPASE
Ref. 13 In-vitro Monkey
Early Amnion
Late Amnion
c
Gel-3D
Ref. 1 Human
Ref. 2 µPASE
Ref. 8 In-vivo Monkey
Ref. 7 In-vitro Monkey
hPSC
MeLC.Day 3
MeLC.Day 8
Epiblast
Primitive streak
Nascent Meso.
Emergent Meso.
MeLC2
MeLC1
PostE-EP
PostL-EPI
Gast1
Gast2a
Gast2b
Mesoderm 2
Mesoderm 1
Ref. 9 Human
Ref. 12 µPASE
Ref. 25 In-vivo Monkey
Ref. 13 In-vitro Monkey
1
0.8
0.6
0.4
0.2
0

Supplementary Figure 5: Heat map of correlation coefficients among indicated *in vivo* and *in vitro* cell clusters.

a. Heat map of correlation coefficients among hPGCLCs derived in the Gel-3D culture on Day 3 (hPGCLC.D3) and on Day 8 (hPGCLC.D8), hPGCs from *in vivo* human gastrula[1], hPGCLCs from microfluidic PASEs[2], hPGCLCs from different *in* vitro protocols[3-5], and gonadal hPGCs (lPGC[4]). Correlation coefficients were calculated based on ontogenic cynomolgus monkey PGC (CyPGC) genes[6] (447 in common out of 544; see **Methods**).

b. Heat map of correlation coefficients among hPSCs, AMLCs derived in the Gel-3D culture on Day 3 (AMLC1.D3 and AMLC2.D3) and on Day 8 (AMLC.D8), AMLCs from microfluidic PASEs[2], amniotic/embryonic ectoderm cells from *in vivo* human gastrula[1], and amnion cells from *in vitro* cultured cynomolgus monkey embryos[7]. Comparisons between indicated cell clusters are based on ontogenic amniotic ectoderm genes identified from *in vitro* cultured cynomolgus monkey embryos (534 in common out of 776; see **Methods**).

c. Heat map of correlation coefficients among hPSCs, MeLCs derived in the Gel-3D culture on Day 3 (MeLC.D3) and on Day 8 (MeLC.D8), MeLCs from microfluidic PASEs[2], mesodermal cells from *in vivo* human gastrula[1], and mesodermal cells from *in vivo* cynomolgus monkey embryo[8] and *in vitro* cultured cynomolgus monkey embryos[7]. Comparisons between indicated cell clusters are based on ontogenic genes identified for the EPI of cynomolgus monkey (575 in common out of 776; see **Methods**).

For DEG list, see **Supplementary Data 1.**

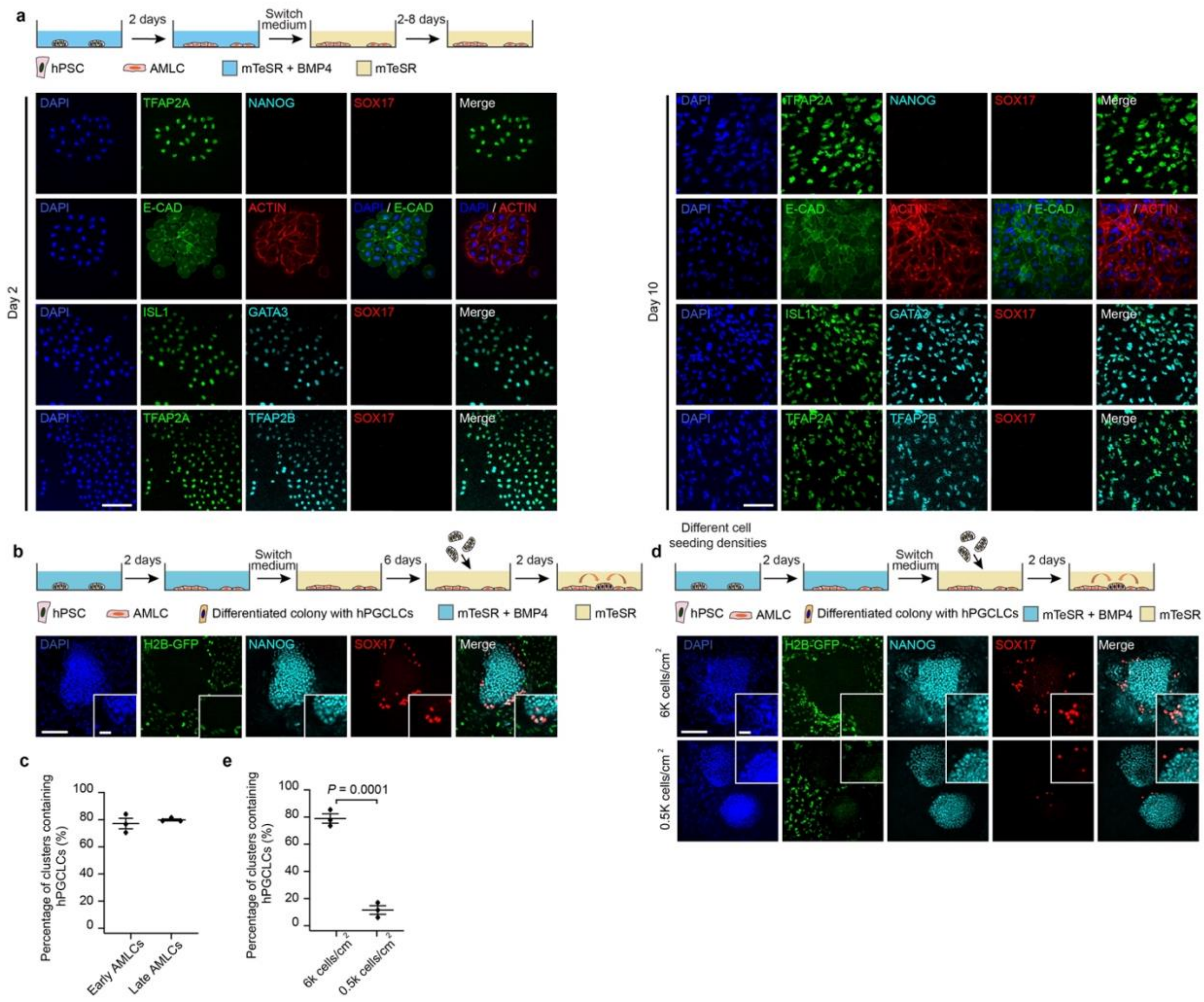

Supplementary Figure 6: Characterization of AMLCs generated in 2D culture system.

a. Derivation of AMLCs by stimulating hPSCs with exogeneous BMP4 (50 ng mL^{-1}) for 48 h (top), after which culture medium was replaced with fresh mTeSR without BMP4 and the cells were cultured for another 8 days. Representative micrographs showing incipient AMLCs stained for TFAP2A, NANOG, and SOX17; ECAD, and ACTIN; ISL1, GATA3, and SOX17; and TFAP2A, TFAP2B, and SOX17 at indicated time points.

b. Co-culture assays of AMLCs and hPSCs. H2B-GFP hPSCs were first differentiated into AMLCs by culturing in mTeSR supplemented with BMP4 (50 ng mL^{-1}) for 48 h. Culture medium was then switched to fresh mTeSR without BMP4 and cultured for another 6

days. On day 8, undifferentiated hPSC clusters were added and co-cultured with AMLCs for 48 h. Representative micrographs showing GFP signal and immunostaining for NANOG and SOX17 in the co-culture on day 10.

c. Plot showing percentages of clusters containing NANOG+SOX17+ hPGCLCs in the co-culture system at indicated conditions.

d. Co-culture assays of AMLCs and hPSCs. H2B-GFP hPSCs with different initial cell seeding densities were first differentiated into AMLCs by culturing in mTeSR supplemented with BMP4 (50 ng mL^{-1}) for 48 h. Culture medium was then switched to fresh mTeSR before undifferentiated hPSC clusters were added and co-cultured with AMLCs for another 2 days. Representative micrographs showing GFP signal and immunostaining for NANOG and SOX17 in the co-culture with different initial cell seeding densities for AMLCs as indicated.

e. Plot showing percentages of clusters containing NANOG+SOX17+ hPGCLCs in the co-culture system at indicated conditions.

In **a-c**, experiments were repeated three times with similar results. Nuclei were counterstained with DAPI. Boxed images in **b & c** show magnified views of selected areas. Scale bars, 100 µm (main panels) and 30 µm (insets). In **c & e**, n = 3 independent experiments, and data represent the mean ± s.e.m. P-value was calculated using unpaired, two-sided Student's t-test. Source data are provided as a Source Data file.

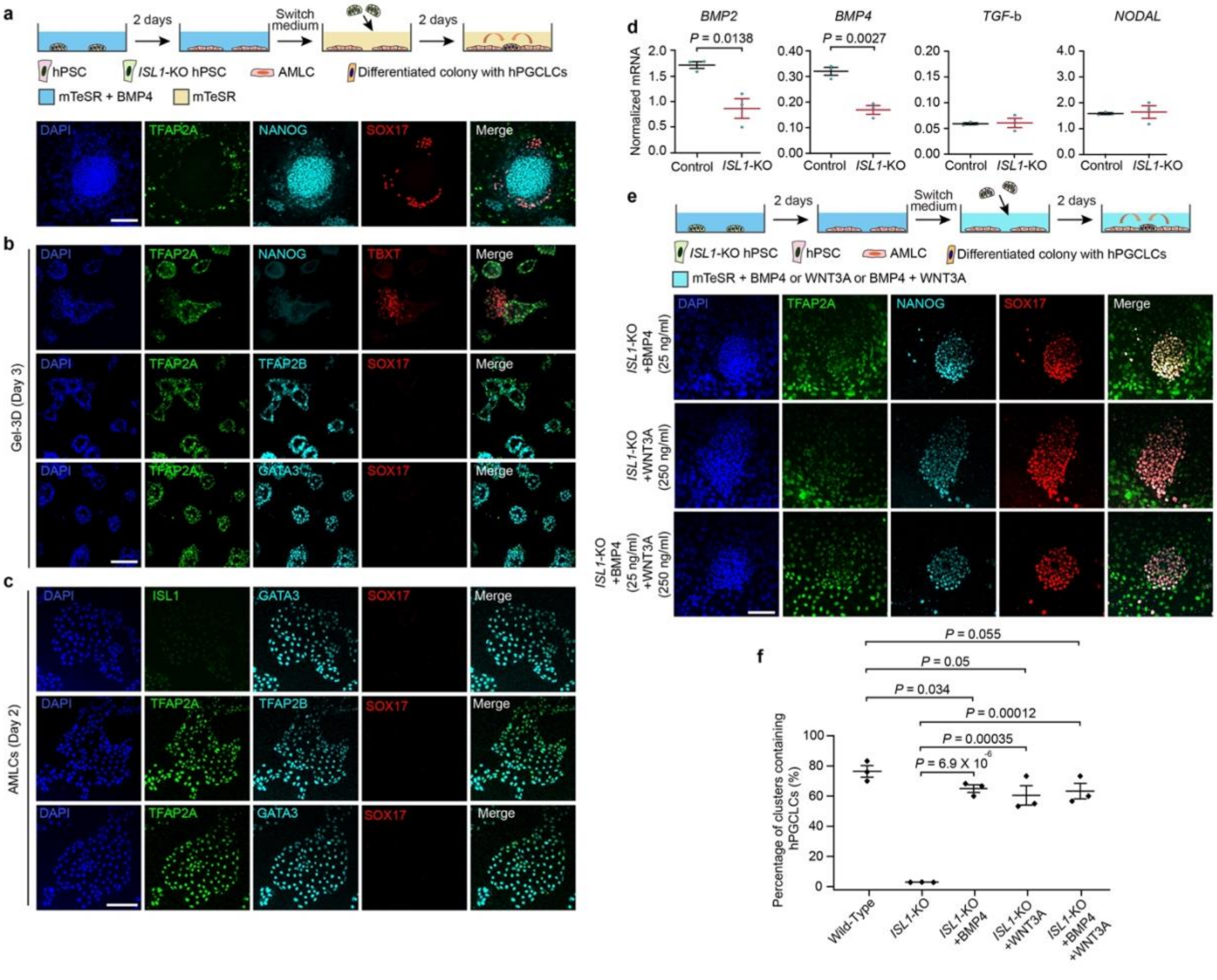

Supplementary Figure 7: Differentiation of *ISL1*-KO hPSCs in both Gel-3D and the co-culture systems.

a. Co-culture assays of AMLCs and *ISL1*-KO hPSCs. Control hPSCs were first differentiated into AMLCs by culturing in mTeSR supplemented with BMP4 (50 ng mL^{-1}) for 2 days. Culture medium was then switched to fresh mTeSR before clusters of *ISL1*-KO hPSCs were added and co-cultured with AMLCs for another 2 days. Micrographs showing immunostaining for TFAP2A, NANOG, and SOX17 on Day 4.

- **b.** Micrographs showing immunostaining of the Gel-3D culture with *ISL1*-KO hPSCs. Cells were stained for TFAP2A, NANOG, and TBXT; TFAP2A, TFAP2B, and SOX17; and TFAP2A, GATA3, and SOX17, on Day 3.
- **c.** Representative micrographs showing incipient AMLCs stained for ISL1, GATA3, and SOX17; TFAP2A, TFAP2B, and SOX17; and TFAP2A, GATA3, and SOX17 at Day 2.
- **d.** RT-PCR analyses showing expression of *BMP2*, *BMP4*, *TGF-β*, and *NODAL* in the Gel-3D culture on Day 3 generated from wild-type and *ISL1*-KO hPSCs as indicated.
- **e.** Co-culture assays of *ISL1*-KO AMLCs and hPSCs. *ISL1*-KO hPSCs were first differentiated into AMLCs by culturing in mTeSR supplemented with BMP4 (50 ng mL^{-1}) for 2 days. Culture medium was then switched to fresh mTeSR supplemented with BMP4 (top), WNT3A (middle), or the combination of both BMP4 and WNT3A (down), as indicated, before undifferentiated hPSC clusters were added and co-cultured with *ISL1*-KO AMLCs for another 2 days. Micrographs showing immunostaining for TFAP2A, NANOG, and SOX17 on Day 4.
- **f.** Plot showing percentages of clusters containing NANOG+SOX17+ hPGCLCs in the co-culture system at indicated conditions.

In **a-c *& e***, experiments were repeated three times with similar results. Nuclei were counterstained with DAPI. Scale bars, 100 µm (main panels) and 30 µm (insets). In **d & f**, n = 3 independent experiments, and data represent the mean ± s.e.m. P-values were calculated using unpaired, two-sided Student's t-test. Source data are provided as a Source Data file.

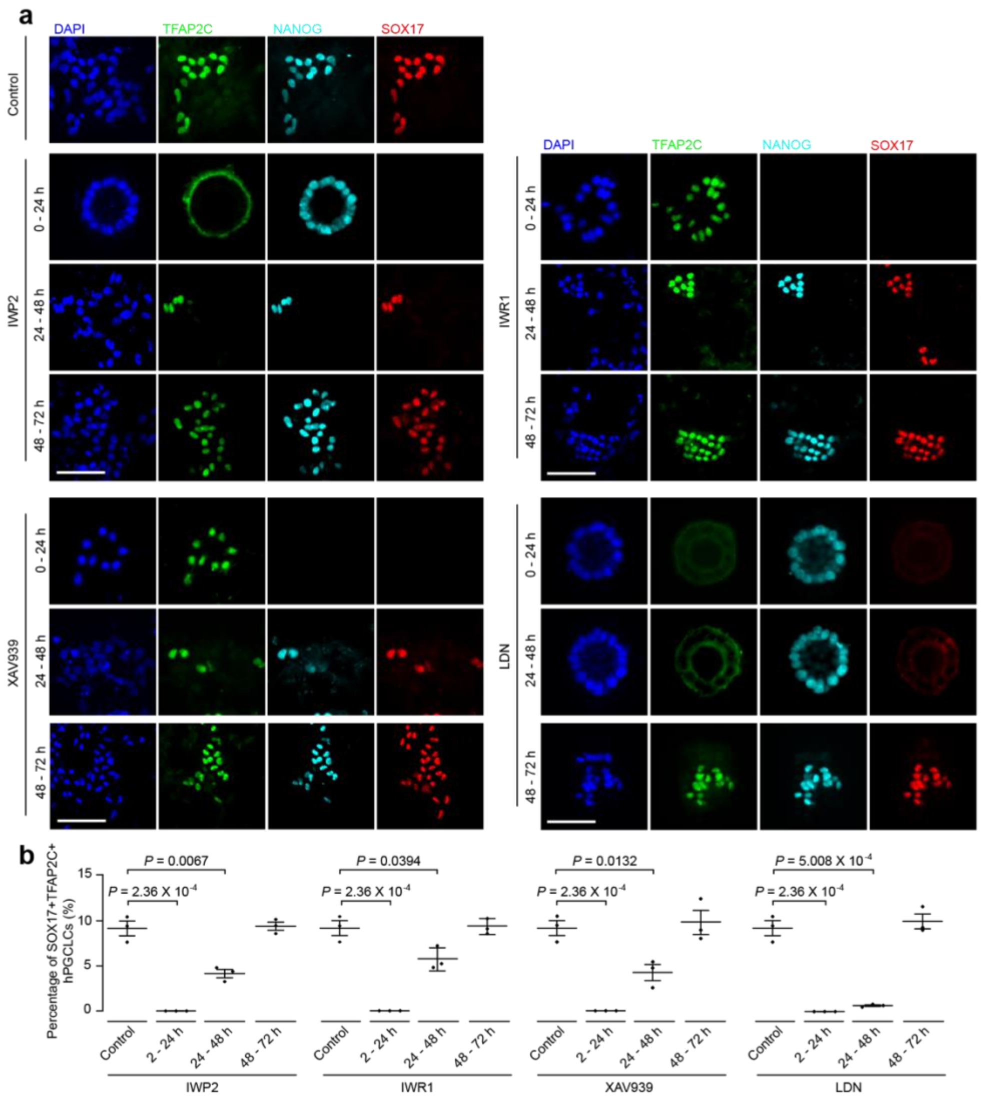

Supplementary Figure 8: hPGCLC induction in the Gel-3D niche is inhibited by IWP2, IWR1, XAV939 or LDN.

- **a.** Micrographs showing immunostaining for TFAP2C, NANOG, and SOX17 for the Gel-3D culture with IWP2, IWR1, XAV939, or LDN supplemented for indicated durations.
- **b.** Plot showing percentages of TFAP2C+SOX17+ hPGCLCs in the Gel-3D culture on Day 3 as a function of IWP2, IWR1, XAV939, or LDN treatments as indicated.

In **a**, experiments were repeated three times with similar result. Nuclei were counterstained with DAPI. Scale bars, 100 μm. In **b**, n = 3 independent experiments, and data represent the mean ± s.e.m. P-value was calculated using unpaired, two-sided Student's t-test. Source data are provided as a Source Data file.

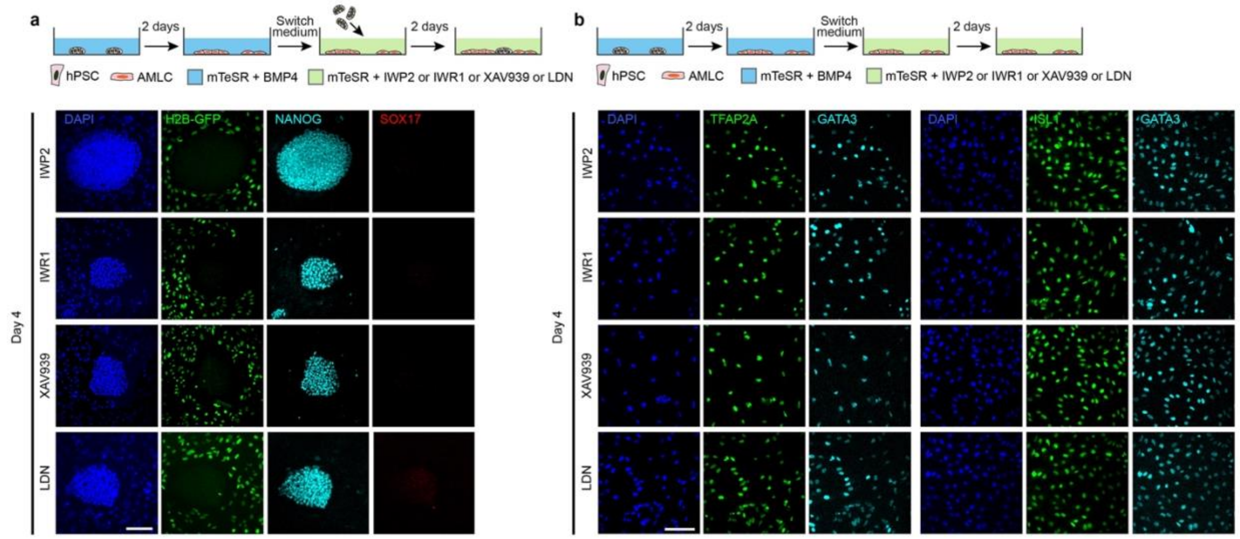

Supplementary Figure 9: hPGCLC induction in the co-culture system is inhibited by IWP2, IWR1, XAV939 or LDN.

- **a.** Schematic shows co-culture assays of AMLCs and hPSCs. H2B-GFP hPSCs were first differentiated into AMLCs by culturing in mTeSR supplemented with BMP4 (50 ng mL^{-1}) for 48 h. Culture medium was then switched to mTeSR supplemented with IWP2, IWR1, XAV939 or LDN, as indicated, before undifferentiated hPSCs were seeded and co-cultured with AMLCs for another 2 days. Representative micrographs showing GFP signals and immunostaining for NANOG and SOX17 in the co-cultures.

b. H9 hPSCs were first differentiated into AMLCs by culturing in mTeSR supplemented with BMP4 (50 ng mL^{-1}) for 48 h. Culture medium was then switched to mTeSR supplemented with IWP2, IWR1, XAV939, or LDN, as indicated and cultured for another 2 days. Representative micrographs at the bottom show immunostaining for TFAP2A, and GATA3; and ISL1, and GATA3 as a function of IWP2, IWR1, XAV939, or LDN treatments as indicated.

In **a & b**, experiments were repeated three times with similar result. Nuclei were counterstained with DAPI. Scale bars, 100 µm.

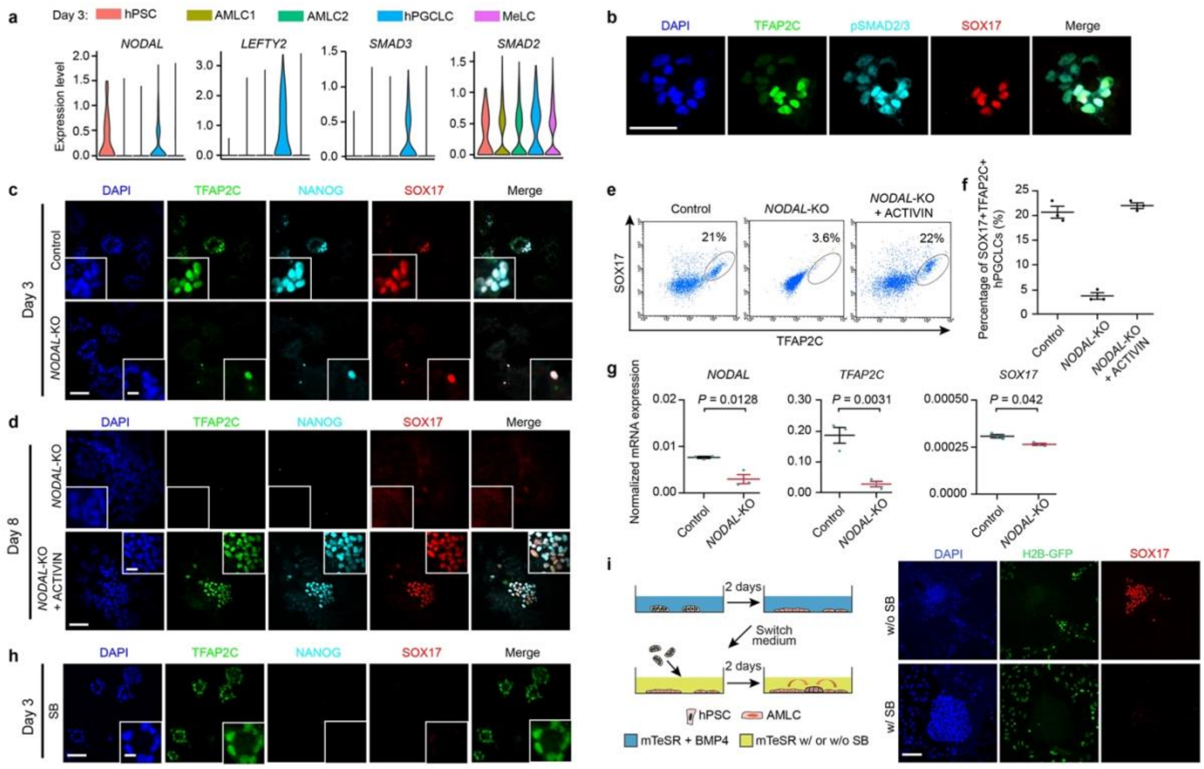

Supplementary Figure 10: Endogenous NODAL signaling is involved in hPGCLC specification in the Gel-3D culture.

- **a.** Violin plots of log-transformed, normalized expression levels of selected NODAL target genes in different cell clusters developed in the Gel-3D culture on Day 3.
- **b.** Micrographs showing immunostaining for TFAP2C, pSMAD2/3, and SOX17 in the Gel-3D culture on Day 3.
- **c.** Micrographs showing immunostaining on Day 3 for TFAP2C, NANOG, and SOX17 in the Gel-3D culture generated from control and *NODAL*-KO hPSCs.
- **d.** Micrographs showing immunostaining on Day 8 for TFAP2C, NANOG, and SOX17 in the Gel-3D culture generated from *NODAL*-KO hPSCs without or with supplementation of ACTIVIN A as indicated.

e. Enumeration by flow cytometry of TFAP2C+SOX17+ hPGCLCs in the Gel-3D culture on Day 8, generated from wild-type control hPSCs, *NODAL*-KO hPSCs, and *NODAL*-KO hPSCs supplemented with ACTIVIN A, as indicated.

f. Plot showing percentages of TFAP2C+SOX17+ hPGCLCs on Day 8 in the Gel-3D culture, generated from wild-type control hPSCs, *NODAL*-KO hPSCs, and *NODAL*-KO hPSCs supplemented with ACTIVIN A, as indicated.

g. RT-PCR analysis of *NODAL, TFAP2C, and SOX17* expression on Day 3 in the Gel-3D culture generated from wild-type and *NODAL*-KO hPSCs.

h. Micrographs showing immunostaining on Day 3 for TFAP2C, NANOG, and SOX17 in the Gel-3D culture supplemented with SB431542 (SB).

i. Co-culture assay of AMLCs and hPSCs with or without supplementation with SB431542 (SB). H2B-GFP hPSCs were first differentiated into AMLCs by BMP4 stimulation (50 ng mL^{-1}) for 48 h. Culture medium was then switched to fresh mTeSR with or without supplementation with SB. Undifferentiated hPSCs were then added and co-cultured with AMLCs for another 48 h. Micrographs show immunostaining on day 4 for SOX17 for the co-culture with or without SB treatment as indicated.

In **b-e**, **h** & **I**, experiments were repeated three times with similar results. In **b-d**, **h** & **i**, nuclei were counterstained with DAPI. Boxed images show magnified views of selected areas. Scale bars, 100 µm (main panels) and 30 µm (insets). In **f** & **g**, n = 3 independent experiments, and data represent the mean ± s.e.m. P-values were calculated using unpaired, two-sided Studen''s t-test. Source data are provided as a Source Data file.

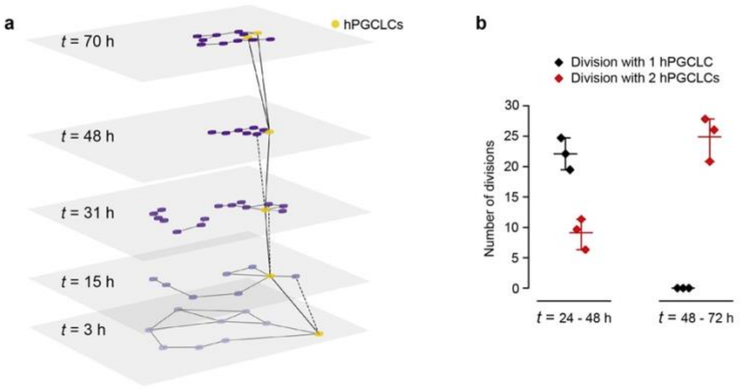

Supplementary Figure 11: hPGCLC specification occurs within two days in the Gel-3D culture.

- **a.** Network representation of hPGCLC development at different timepoints in the Gel-3D culture. Each cylinder represents a cell in the culture. Connections between cells are established based a threshold distance. Cell lineage of yellow-colored cells, representing hPGCLC lineage, is shown with black lines connecting related cells at consecutive time points. Intensity of color reflects different time points.
- **b.** Plot showing number of cell divisions, leading to either one hPGCLC or two hPGCLCs at different days. n = 3 independent experiments. Data represent the mean ± s.e.m. Source data are provided as a Source Data file.

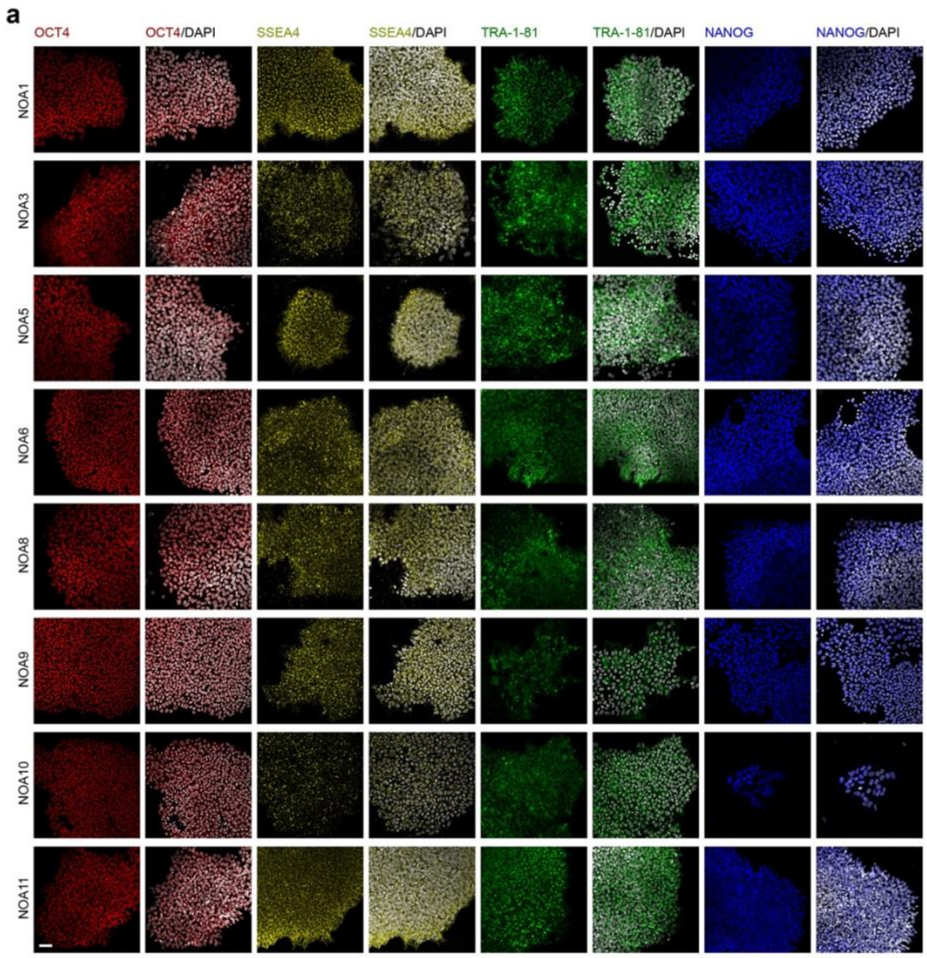

Supplementary Figure 12: Immunofluorescence analysis for self-renewal markers for all NOA hPSC lines. Micrographs showing immunostaining for OCT4, SSEA4, TRA-1-81, and NANOG. Experiments were repeated three times with similar result. Nuclei were counterstained with DAPI. Scale bars, 50 µm.

Supplementary Table 1: List of primary antibodies.

Targeted Protein	Species	Dilution	Catalog No.	Vendor
EZRIN	Mouse	1:2000	E8897	Sigma-Aldrich
E-CADHERIN	Mouse	1:500	610181	BD Biosciences
ACTIN	Goat	1:200	LS-B15553	Lifespan Bioscience
OCT4	Mouse	1:200	SC-5279	Santa-Cruz Biotechnology
NANOG	Rabbit	1:500	4903S	Cell Signaling Technology
NANOG	Goat	1:100	ab109250	Abcam
TFAP2A	Mouse	1:100	sc-12726	Santa-Cruz Biotechnology
TFAP2C	Mouse	1:100	sc-12762	Santa-Cruz Biotechnology
BRACHYURY	Rabbit	1:200	81694S	Cell Signaling Technology
GATA3	Rabbit	1:200	D13C9	Cell Signaling Technology
pSMAD2/3	Rabbit	1:100	D27F4	Cell Signaling Technology
SOX17	Goat	1:500	AF1924	R&D Systems
TFAP2B	Rabbit	1:200	2509	Cell Signaling Technology
BLIMP1	Rabbit	1:200	CS#9115S	Cell Signaling Technology
KLF4	Mouse	1:200	SC-166238	Santa-Cruz Biotechnology
VIM	Rabbit	1:200	Ab8978	Abcam
TFCP2L1	Goat	1:200	AF5726-SP	R&D Systems
ISL1	Mouse	1:200	SC-166238	Santa-Cruz Biotechnology

Supplementary Table 2: List of primers used in qRT-PCR. Primers for SYBR green PCR were designed using NCBI-primer BLAST. Sequences were as follows:

Gene	Forward	Reverse
BMP2	ACTACCAGAAACGAGTGGGAA	GCATCTGTTCTCGGAAAACCT
BMP4	AAAGTCGCCGAGATTCAGGG	GACGGCACTCTTGCTAGGC
WNT5a	AGGAGTTCGTGGACGCTAGA	ACTTCTCCTTGAGGGCATCG
WNT5b	ACGCTGGAGATCTCTGAGGA	CGAGGTTGAAGCTGAGTTCC
WNT6	GCGGAGACGATGTGGACTTC	ATGCACGGATATCTCCACGG
TGF-B	GGGCTACCATGCCAACTTCT	GACACAGAGATCCGCAGTCC
NODAL	CATTGCCTCAGGCTGGGTTG	GTACAGCTCATTAGCAGAGAACCA
EOMES	AAGGGGAGAGTTTCATCATCCC	GGCGCAAGAAGAGGATGAAATAG
SOX17	AGTGACGACCAGAGCCAGAC	CCTTAGCCCACACCATGAAA
TFAP2C	ATTAAGAGGATGCTGGGCTCTG	CACTGTACTGCACACTCACCTT

Supplementary Table 3: Summary of patient-derived hiPSC lines used in this study.

Sample ID	Non Obstructive Azoospermia Diagnosis	Fibroblast Karyotype	hiPSC Karyotype	OCT4/ NANOG Expression Y/N?	SSEA4/ TRA-1-81 Expression Y/N?
NOA1	Maturation arrest	Normal	Normal	Yes	Yes
NOA3	Very Severe Oligospermia	Ch8 +p23.2	Ch8 +p23.2	Yes	Yes
NOA5	SCOS	Normal	Normal	Yes	Yes
NOA6	SCOS	Normal	Normal	Yes	Yes
NOA8	SCOS	Normal	Normal	Yes	Yes
NOA9	SCOS	Normal	Normal	Yes	Yes
NOA10	SCOS	Normal	Normal	Yes	Yes
NOA11	Maturation arrest	Normal	Normal	Yes	Yes

Supplementary References:

1 Tyser, R. C. V. *et al.* Single-cell transcriptomic characterization of a gastrulating human embryo. *Nature* **600**, 285-289 (2021). https://doi.org:10.1038/s41586-021-04158-y
2 Zheng, Y. *et al.* Controlled modelling of human epiblast and amnion development using stem cells. *Nature* **573**, 421-425 (2019).
3 Sasaki, K. *et al.* Robust in vitro induction of human germ cell fate from pluripotent stem cells. *Cell stem cell* **17**, 178-194 (2015).
4 Irie, N. *et al.* SOX17 is a critical specifier of human primordial germ cell fate. *Cell* **160**, 253-268 (2015).
5 Chen, D. *et al.* Human primordial germ cells are specified from lineage-primed progenitors. *Cell reports* **29**, 4568-4582. e4565 (2019).
6 Sasaki, K. *et al.* The germ cell fate of cynomolgus monkeys is specified in the nascent amnion. *Developmental cell* **39**, 169-185 (2016).
7 Yang, R. *et al.* Amnion signals are essential for mesoderm formation in primates. *Nature communications* **12**, 1-14 (2021).
8 Nakamura, T. *et al.* A developmental coordinate of pluripotency among mice, monkeys and humans. *Nature* **537**, 57-62 (2016).
