# Supplementary material for: Derivation of human primordial germ cell-like cells in an embryonic-like culture
Source: Nat Commun. 2024 Jan 2;15:167. doi: 10.1038/s41467-023-43871-2 (PMC10762101; doi:10.1038/s41467-023-43871-2)
Supplement: Supplementary file 3 — Description of Additional Supplementary Files [file 41467_2023_43871_MOESM3_ESM.pdf]

## Description of Additional Supplementary Files

**Supplementary Data 1 :** Ontogenic genes and DEG lists.

**Supplementary Movie 1 : Development of columnar epiblast-like cysts and squamous amniotic ectoderm-like cysts in the Gel-3D culture.** Representative time-lapse video showing progressive development of hPSC-derived epithelial cysts with different morphologies (columnar and squamous cysts) in the Gel-3D culture. Time stamps indicate culture time (t). Scale bar, 50 µm. Experiments were repeated three times with similar results.

**Supplementary Movie 2. : Development of irregular cysts in the Gel-3D culture.** Representative time-lapse video showing progressive development of hPSC-derived irregular cyst in the Gel-3D culture. After finishing live imaging, cells were stained for DAPI and hPGCLC markers (TFAP2C, NANOG, and SOX17) to detect the presence of hPGCLCs in the irregular cyst. Time stamps indicate culture time (t). Scale bar, 50 µm. Experiments were repeated five times with similar results.
