# Supplementary material for: Derivation of human primordial germ cell-like cells in an embryonic-like culture
Source: Nat Commun. 2024 Jan 2;15:167. doi: 10.1038/s41467-023-43871-2 (PMC10762101; doi:10.1038/s41467-023-43871-2)
Supplement: Supplementary file 7 — Reporting Summary [file 41467_2023_43871_MOESM7_ESM.pdf]

nature portfolio

Corresponding author(s): Jianping Fu
Amander Clark

Last updated by author(s): Nov 11, 2023

Reporting Summary

Nature Portfolio wishes to improve the reproducibility of the work that we publish. This form provides structure for consistency and transparency in reporting. For further information on Nature Portfolio policies, see our Editorial Policies and the Editorial Policy Checklist.

Statistics

For all statistical analyses, confirm that the following items are present in the figure legend, table legend, main text, or Methods section.

n/a	Confirmed	
☐	☒	The exact sample size (*n*) for each experimental group/condition, given as a discrete number and unit of measurement
☐	☒	A statement on whether measurements were taken from distinct samples or whether the same sample was measured repeatedly
☐	☒	The statistical test(s) used AND whether they are one- or two-sided<br>*Only common tests should be described solely by name; describe more complex techniques in the Methods section.*
☐	☒	A description of all covariates tested
☐	☒	A description of any assumptions or corrections, such as tests of normality and adjustment for multiple comparisons
☐	☒	A full description of the statistical parameters including central tendency (e.g. means) or other basic estimates (e.g. regression coefficient) AND variation (e.g. standard deviation) or associated estimates of uncertainty (e.g. confidence intervals)
☐	☒	For null hypothesis testing, the test statistic (e.g. *F*, *t*, *r*) with confidence intervals, effect sizes, degrees of freedom and *P* value noted<br>*Give P values as exact values whenever suitable.*
☒	☐	For Bayesian analysis, information on the choice of priors and Markov chain Monte Carlo settings
☒	☐	For hierarchical and complex designs, identification of the appropriate level for tests and full reporting of outcomes
☒	☐	Estimates of effect sizes (e.g. Cohen's *d*, Pearson's *r*), indicating how they were calculated

Our web collection on statistics for biologists contains articles on many of the points above.

Software and code

Policy information about availability of computer code

Data collection	MetaMorph Advanced, AxioVision, Cell Ranger
Data analysis	MATLAB, ImageJ and Excel (Microsoft), RStudio, Seurat package, Guava® InCyte, Prism

For manuscripts utilizing custom algorithms or software that are central to the research but not yet described in published literature, software must be made available to editors and reviewers. We strongly encourage code deposition in a community repository (e.g. GitHub). See the Nature Portfolio guidelines for submitting code & software for further information.

Data

Policy information about availability of data

All manuscripts must include a data availability statement. This statement should provide the following information, where applicable:

- Accession codes, unique identifiers, or web links for publicly available datasets
- A description of any restrictions on data availability
- For clinical datasets or third party data, please ensure that the statement adheres to our policy

Data supporting the findings of this study are available within the article and its supplementary information files and from the corresponding authors upon reasonable request.

Research involving human participants, their data, or biological material

Policy information about studies with human participants or human data. See also policy information about sex, gender (identity/presentation), and sexual orientation and race, ethnicity and racism.

Reporting on sex and gender	All the participants are male. The participants are diagnosed with non-obstructive azoospermia (NOA) which is a disease only for men.
Reporting on race, ethnicity, or other socially relevant groupings	All the information has been provided in Supplementary Information (Supplementary Table 4).
Population characteristics	All the information has been provided in Supplementary Information (Supplementary Table 4).
Recruitment	There is no potential self-selection bias or other biases for the recruitment of participants.
Ethics oversight	This study was approved and reviewed annually by the UCLA Institutional Review Board (IRB #18-001466) together with annual review by the UCLA Human Pluripotent Stem Cell Research and Oversight (hPSCRO) Committee (hPSCRO #2018-005-04).

Note that full information on the approval of the study protocol must also be provided in the manuscript.

Field-specific reporting

Please select the one below that is the best fit for your research. If you are not sure, read the appropriate sections before making your selection.

☒ Life sciences ☐ Behavioural & social sciences ☐ Ecological, evolutionary & environmental sciences

For a reference copy of the document with all sections, see nature.com/documents/nr-reporting-summary-flat.pdf

Life sciences study design

All studies must disclose on these points even when the disclosure is negative.

Sample size	All experiments were conducted with at least two independent experiments and multiple biological replicates. Sample sizes were determined based on our previous experience and similar studies of other groups. Sample sizes were determined as sufficient since they led to similar results.
Data exclusions	Due to intrinsic inhomogeneity of Geltrex, some Geltrex continued to contract during experiments. Such experiments were excluded from data analysis.
Replication	Reported results were repeated and confirmed for at least two independent experiments. Key experimental findings were reliably reproduced by two investigators involved in this work.
Randomization	Samples were randomly allocated to control and different experimental groups (see Methods). However, no particular randomization method was used in this work.
Blinding	Investigators were not blinded to group allocation.

Reporting for specific materials, systems and methods

We require information from authors about some types of materials, experimental systems and methods used in many studies. Here, indicate whether each material, system or method listed is relevant to your study. If you are not sure if a list item applies to your research, read the appropriate section before selecting a response.

Materials & experimental systems

n/a	Involved in the study	
☐	☒	Antibodies
☐	☒	Eukaryotic cell lines
☒	☐	Palaeontology and archaeology
☒	☐	Animals and other organisms
☒	☐	Clinical data
☒	☐	Dual use research of concern
☒	☐	Plants

Methods

n/a	Involved in the study	
☒	☐	ChIP-seq
☐	☒	Flow cytometry
☒	☐	MRI-based neuroimaging

Antibodies

Antibodies used	Antibody information (including species, application, and catalog number) has been provided in Supplementary Information (Supplementary Table 2).
Validation	All antibodies have been validated by the companies from which they were purchased from. The subcellular localization of all the proteins analyzed in this work has been previously reported. This information was used to further validate the specificity of antibodies.

Eukaryotic cell lines

Policy information about cell lines and Sex and Gender in Research

Cell line source(s)	The following cell lines were used in this work:<br>H9 hESC line (WA09, WiCell; NIH registration number: 0062); H1 hESC line (WA01, WiCell; NIH registration number: 0043); HES-3 hESC (WiCell); 1196a line (human induced pluripotent stem cell, or hiPSC, from the University of Michigan Pluripotent Stem Cell Core); and nine hiPSC lines derived from research participants diagnosed with NOA.
Authentication	All hPSC lines have been authenticated by the original sources and also authenticated in-house by immunostaining for pluripotency markers and successful differentiation to the three germ layer cells.
Mycoplasma contamination	All cell lines used in this work have been tested negative for mycoplasma contamination.
Commonly misidentified lines<br>(See ICLAC register)	No commonly misidentified cell lines listed by ICLAC were used in this work.

Flow Cytometry

Plots

Confirm that:

☒ The axis labels state the marker and fluorochrome used (e.g. CD4-FITC).

☒ The axis scales are clearly visible. Include numbers along axes only for bottom left plot of group (a 'group' is an analysis of identical markers).

☒ All plots are contour plots with outliers or pseudocolor plots.

☒ A numerical value for number of cells or percentage (with statistics) is provided.

Methodology

Sample preparation	Samples from different days were treated with Accutase for 1 h to obtain single-cell suspensions. Single cells were then fixed in 4% PFA for 1 h and then permeabilized in 0.2% Triton X-100 (Roche Applied Science) for 30 min. Samples were then blocked in 2% BSA for 1 h and then incubated with primary antibody for 1 h. Samples were then labeled with donkey-raised secondary antibodies (1:400) for another 1 h.
Instrument	Guava EasyCyte (Luminex) flow cytometer
Software	Guava® InCyte Software
Cell population abundance	We were trying to find the percentage of hPGCLCs in the population. It was about 10% by day 3 and 20% by day 8.
Gating strategy	In the control experiment, we made the boundaries for the separated cluster to show the cell population that we are interested. The same boundaries were used for the rest of the experiments.

☒ Tick this box to confirm that a figure exemplifying the gating strategy is provided in the Supplementary Information.
